# Pruritus in intrahepatic cholestasis of pregnancy: the role of substance p

**DOI:** 10.1590/1806-9282.20242025

**Published:** 2025-06-16

**Authors:** Ömer Gökhan Eyisoy, Çağdaş Özgökçe, Ümit Taşdemir, Sinem Tekin, Oya Demirci

**Affiliations:** 1 Zeynep Kamil Women and Children Diseases Training and Research Hospital Affiliated with the University of Health Sciences, Department of Obstetrics, Division of Perinatology – İstanbul, Turkey.; 2 Haseki Training and Research Hospital Affiliated with the University of Health Sciences, Department of Obstetrics and Gynecology – İstanbul, Turkey.

**Keywords:** Substance P, Pruritus, Itch, Cholestasis, Pregnancy

## Abstract

**OBJECTIVE:**

The aim of this study was to evaluate the role of substance P in pruritus in patients with intrahepatic cholestasis of pregnancy.

**METHODS:**

This prospective case–control study was conducted on 50 intrahepatic cholestasis of pregnancy patients and 30 gestational age-matched healthy pregnant women. The groups were compared in terms of serum substance P concentrations, and the correlation between these concentrations and self-reported itching was evaluated in patients with intrahepatic cholestasis of pregnancy.

**RESULTS:**

Serum substance P concentrations were higher in patients with intrahepatic cholestasis of pregnancy than in the control group (960 vs. 611 ng/L; p=0.001). In intrahepatic cholestasis of pregnancy patients, there was a statistically significant positive correlation between the serum substance P concentration and both the Pruritus Severity Scale score (r=605, p<0.001) and the visual analog scale score for pruritus (r=483, p<0.001). On multivariable linear regression analysis, only the presence of intrahepatic cholestasis of pregnancy (pruritus) was statistically significantly associated with an increase in the serum substance P concentration (95%CI 145.06–558.16 ng/L; p=0.001).

**CONCLUSION:**

The concentration of substance P is increased in patients with intrahepatic cholestasis of pregnancy and is correlated with the severity of the pruritus. While the precise role of elevated substance P levels in the etiology of intrahepatic cholestasis of pregnancy remains uncertain, it may contribute to the pathogenesis of pruritus through central and peripheral effects.

## INTRODUCTION

Intrahepatic cholestasis of pregnancy (ICP) is the most common pregnancy-specific liver disease. Although the importance of ICP is attributable to adverse fetal effects such as low Apgar scores at birth, meconium-stained amniotic fluid, preterm labor, and intrauterine fetal death, maternal presentation is usually with pruritus. Pruritus typically starts on the palms of the hands and soles of the feet and is more intense in these areas but can also be widespread. Although a rash specific to ICP is not observed (a rash may be indicative of a dermatological disease), scratch marks and abrasions of the skin may be observed depending on the patient's response to pruritus^1^.

Pruritus, or itch, can be described as an irritating sensation on the skin with an immediate need to scratch^2^. Histaminergic and non-histaminergic pathways have been shown to be involved in the perception and transmission of itch^3^. Although both pathways are important in the transmission of itch signals, the non-histaminergic pathway appears to play a more important role in conditions associated with chronic pruritus^4^.

Substance P (SP) belongs to the tachykinin family and works as a neurotransmitter or modulator. It is produced and secreted by nerve fibers of both the central and peripheral nervous systems and binds to the neurokinin 1 receptor (NK_1_R). NK_1_R is expressed in many skin cells involved in the initiation and induction of pruritus, such as keratinocytes, fibroblasts, and mast cells^5^. SP is associated with itch through the induction of the keratinocyte expression of the nerve growth factor and leukotriene B_4_ and the degranulation of mast cells^6,7^. SP and NK_1_R are also widely expressed in the peripheral sensory nerve fibers and central nervous system, particularly in spinal interneuron functioning in the transmission of the itch signal to the brain^8,9^.

The role of SP has been investigated in many chronic pruritic conditions. However, it has not been studied in patients with ICP. In this context, the aim of this pilot study was to evaluate the role of SP in pruritus in patients with ICP.

## METHODS

This was a prospective cohort study conducted at *Zeynep Kamil Women and Children Diseases Training and Research Hospital*. Approval for the study was obtained from the Institutional Ethics Committee (date: 05.05.2021, no. 101), and it was carried out in accordance with the principles of the Declaration of Helsinki. Prior to enrollment, written informed consent was obtained from all participants.

### Study design

In the period between January 2022 and January 2024, 216 pregnant women who were complaining of pruritus in the absence of any dermatological conditions or rash underwent testing with a fasting serum total bile acid (TBA) level. ICP was diagnosed with fasting serum TBA >10 mmol/L. Patients with known renal disease, hepatobiliary disease, skin disease, or a skin lesion (other than a scratch) with pruritus were not included. Pregnancies with complications such as multiple gestation, gestational diabetes, fetal growth restriction, and hypertensive disorders were excluded. A total of 50 patients diagnosed with ICP were included in the study group. The control group consisted of gestational age-matched healthy singleton pregnancies.

### Pruritus assessment

Pruritus intensity was assessed in two ways: a 12-item Pruritus Severity Scale (PSS) and a visual analog scale (VAS). The PSS is a questionnaire-based scoring system consisting of 12 questions about the intensity, extent, duration, impact on daily life, and scratching response of pruritus. The total score ranges from 3 (the lowest pruritus intensity) to 22 points (the highest pruritus intensity)^10^. The VAS is also a measuring instrument with a horizontal line numbered from 0 (no pruritus) to 10 (worst pruritus). Patients were asked to mark the number that reflected the severity of their pruritus. Both questionnaires were applied at the time of diagnosis.

### Sample collection

All laboratory tests, except SP, were performed according to a protocol for routine blood testing in pregnant patients and were measured in our clinical laboratory. Once the diagnosis of ICP was confirmed, a blood sample was taken to measure the concentration of SP before any treatment was started on the patient. Blood samples collected in gel barrier tubes were centrifuged at 2,500×*g* for 10 min and serum samples were stored at −80°C until analysis. Serum SP concentrations were measured using an enzyme-linked immunosorbent assay (ELISA) kit according to the instructions of the manufacturer (Bioassay Technology Laboratory, Jiaxing, Zhejiang, China) (sensitivity=2.39 ng/L). Concentrations were reported in ng/L.

### Statistics

All statistical analyses were performed using IBM SPSS Statistics version 22.0 (IBM Corporation, Armonk, New York, USA). Variables with a normal distribution were compared using the independent samples t-test. The Mann-Whitney U test was used to compare variables with a non-normal distribution. Spearman's rank correlation was used to assess the association between quantitative variables. Multiple linear regression analysis was performed to determine the relationship between the presence of ICP and SP levels, controlling for potential confounders. The receiver operating characteristic (ROC) curve was used to determine the effectiveness of SP in predicting ICP and its significant threshold. In all statistical analyses, the significance level (p-value) was set at 0.05. No sample size calculation was performed as this was a pilot study.

## RESULTS

A total of 80 patients were enrolled in the study, including 50 patients with ICP and 30 healthy pregnancies (control group). Table 1 shows the clinical characteristics of the groups. Age, parity, body mass index (BMI), and gestational age at enrollment were similar in both groups. Hepatic transaminases and total bilirubin levels were higher in the ICP group than in the control group (p<0.001). Gestational age at delivery and the birth weight of the fetus were lower in the ICP group compared with the control group (p<0.001). Statistically significantly higher concentrations of SP were found in ICP patients than in the control group (960.07 vs. 611.57 ng/L; p<0.001).

**Table 1:** Demographic and clinical characteristics of intrahepatic cholestasis of pregnancy and control groups

	ICP (n=50)	Control (n=30)	p
Age (years)†	29.64±5.82	30.33±6.08	0.614
Parity§	1 (0–2)	1 (0–2)	0.589
BMI (kg/m^2^)†	27.94±3.54	28.35±3.92	0.632
GA at enrollment (weeks)§	34 (32–36)	34 (32–36)	0.571
Fasting total bile acid (mmol/L)	21.0 (13.8–36.0)	–	N/A
AST (U/L)§	55.5 (36.5–106.5)	27.5 (23.7–31.0)	<0.001
ALT (U/L)§	69.0 (45.7–134.0)	26.0 (21.7–28.0)	<0.001
Total bilirubin§	0.51 (0.37–0.61)	0.31 (0.22–0.40)	<0.001
GA at birth (weeks)§	37 (37–38)	39 (39–40)	<0.001
Birth weight (g)§	3,040 (2,865–3,220)	3,270 (3,120–3,615)	<0.001
VAS score	3 (4–5)	–	N/A
PSS score	9 (7–11)	–	N/A
Substance P (ng/L)	960.07±507.97	611.57±325.92	0.001

† Independent samples t-test,

§ Mann-Whitney U test. Values are presented as mean±SD or median (1st–3rd quartiles). ICP: intrahepatic cholestasis of pregnancy; BMI: body mass index; GA: gestational age; AST: aspartate aminotransferase; ALT: alanine aminotransferase; VAS: visual analog scale; PSS: 12-Item Pruritus Severity Scale.

The results of Spearman's correlation analysis indicated a statistically significant positive correlation between the serum SP concentration and both the PSS score (r=605, p<0.001) and the VAS score for pruritus (r=483, p<0.001). No statistically significant correlation was observed between the PSS or VAS for pruritus and fasting bile acid, hepatic transaminases, or total serum bilirubin levels (p>0.05).

Multiple linear regression analyses indicated that when controlled for maternal age, gestational age, BMI, and multiparity, only the presence of ICP (presence of pruritus) was found to be significantly associated with an increase in the serum SP concentration. Patients with ICP were likely to have a 351.61 ng/L increase in the serum SP concentration (95%CI 145.06–558.16 ng/L; p=0.001) (Table 2).

**Table 2:** Multiple linear regression model

Variables	Estimate±SE	95% confidence interval	p-value
Presence of ICP (pruritus)	351.61±103.66	145.06–558.16	0.001
Maternal age	-12.31±8.60	-29.47 to 4.83	0.157
GA at diagnosis	11.14±19.24	-27.20 to 49.49	0.564
Body mass index	8.75±13.76	-18.66 to 36.17	0.527
Multiparity	165.01±108.20	-50.58 to 380.61	0.132

SE: standard error; ICP: ıntrahepatic cholestasis of pregnancy; GA: gestational age.

**Figure 1:**
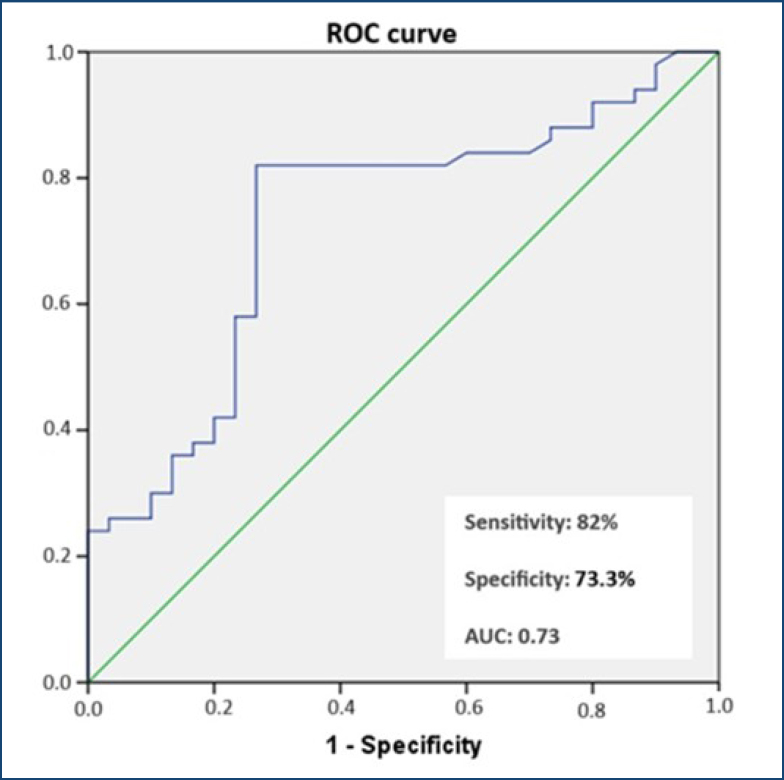
Receiver operating characteristic curve for substance P in predicting intrahepatic cholestasis of pregnancy; optimal cutoff=562 ng/L.

A ROC curve analysis was conducted to identify the optimal cutoff value for SP levels in predicting ICP. The optimal threshold value for serum SP concentration was determined as 562 ng/L with 82% sensitivity and 73.3% specificity. The area under the curve (AUC) was 0.730 (95%CI 0.614–0.847, p<0.01) (Figure 1).

## DISCUSSION

The pathogenesis of pruritus in cholestasis is still not well understood. The bile acid theory suggests that increased skin bile acid levels are pruritogenic^11^. However, the absence of pruritus in some patients with cholestasis despite elevated plasma bile acid levels and the lack of an apparent correlation between cutaneous bile acid concentrations in patients with chronic cholestasis and the presence or severity of pruritus are not consistent with this theory^12^. In this study, we also found no correlation between the severity of pruritus and plasma bile acid levels in patients with ICP.

SP and its receptor NK_1_R are perhaps not the most important but one of the best-characterized neuropeptides in the pathogenesis of pruritus^2^. Many studies indicate an increase in the number of nerve fibers expressing SP in itchy skin, as well as an increase in SP expression in nerve fibers in proximity to the dermoepidermal junction. Furthermore, overexpression of the NK_1_R in the epidermis has been demonstrated^13,14^. An elevated plasma concentration of SP has also been reported in dermatological conditions characterized by pruritus, including atopic dermatitis and psoriasis^15,16^.

Investigations have also been conducted into the potential role of SP in the context of non-dermatological chronic diseases characterized by pruritus. It was reported that plasma SP concentrations were higher in patients with renal pruritus compared to the control group^17^. Koyuncu et al. demonstrated that the severity of pruritus in dialysis patients was proportional to the quantity of SP^18^. In contrast to these findings, Snit et al. reported that there was no difference in plasma SP levels between peritoneal dialysis and hemodialysis patients and that SP did not contribute to pruritus in dialysis patients. It should be noted that no control group was employed in this study, and therefore a comparison between dialysis patients with pruritus and a healthy population was not carried out^19^.

A review of the literature reveals a paucity of studies examining the role of SP in liver diseases. Trivedi et al. showed that patients with chronic liver disease and pruritus had higher plasma levels of SP than patients with chronic liver disease without itching and the control group. Furthermore, the study demonstrated that the plasma concentration of SP in rats with cholestasis secondary to bile duct resection was elevated in comparison to the sham resected control group. Hence, they suggested a potential role of SP in liver disease and in the mediation of pruritus^20^. In the current study, we found higher serum concentrations of SP in patients with ICP than in healthy subjects, even after controlling for potential confounders. Additionally, a positive correlation was observed between serum SP levels and the severity of pruritus.

Increasing evidence suggests that endogenous opioids play an important role in cholestatic pruritus. Activation of μ-opioid receptors by elevated endogenous opioids in chronic liver disease has been implicated in the pathogenesis of pruritus^21^. In rat models, the administration of μ-opioid receptor antagonists or κ-opioid receptor agonists was demonstrated to be an effective method for reducing the scratching behavior induced by an injection of SP^22^. The data suggest that SP may be involved in the mediation of cholestatic pruritus via the opioid system.

Despite the ongoing research into the potential fetal effects of ursodeoxycholic acid (UDCA), it has proven efficacy in improving maternal outcomes, including pruritus^23,24^. In some cases, pruritus in ICP may not respond effectively to UDCA. It has been suggested that a combination of medications, including rifampin, dexamethasone, and 5-hydroxytryptamine 3 receptor agonists (e.g., ondansetron), could be effective in the treatment of ICP and/or pruritus associated with it. However, the effectiveness of these proposed therapeutic interventions is limited^1^. NK_1_R antagonism has been identified as a promising target in the treatment of SP-mediated itch^25^. The current study represents a preliminary investigation into the role of SP in patients diagnosed with ICP. This could result in future studies designed to confirm the role of SP in the pathogenesis of pruritus in ICP patients and to assess the efficacy of topical or systemic NK_1_R antagonists, such as aprepitant and serlopitant, in providing relief from pruritus.

This study is not without limitations. First, as this is a pilot study, we were unable to ascertain the optimal sample size. Nevertheless, a post hoc power calculation, based on the available data, demonstrated that the study had 93% power (alpha=0.05, two-tailed) to detect a difference in the plasma SP concentration between patients with ICP and those without it. Second, the expression of NK_1_R or SP was not evaluated in the pruritic skin of patients with ICP.

## CONCLUSION

The current study has shown that the concentration of SP is increased in patients with ICP and is correlated with the severity of the pruritus. While the precise role of elevated SP levels in the etiology of ICP remains uncertain, it may contribute to the pathogenesis of pruritus through central and peripheral effects. It would be beneficial to conduct future studies to evaluate cutaneous SP and NK_1_R expression by immunohistochemistry.
